# Draft Genome Sequence of *Herpotrichiellaceae* sp. UM 238 Isolated from Human Skin Scraping

**DOI:** 10.1128/genomeA.00148-12

**Published:** 2013-02-14

**Authors:** Kee Peng Ng, Su Mei Yew, Chai Ling Chan, Ruixin Tan, Tuck Soon Soo-Hoo, Shiang Ling Na, Hamimah Hassan, Yun Fong Ngeow, Chee-Choong Hoh, Kok Wei Lee, Wai-Yan Yee

**Affiliations:** a Tropical Infectious Diseases Research and Education Centre (TIDREC), Department of Medical Microbiology, University Malaya Medical Centre, Faculty of Medicine, University of Malaya, Kuala Lumpur, Malaysia; b Codon Genomics SB, Jalan Bandar Lapan Belas, Pusat Bandar Puchong, Selangor Darul Ehsan, Malaysia

## Abstract

*Herpotrichiellaceae* spp. are known to be opportunistic human pathogens. Here, we report the ~28.46-Mb draft genome of *Herpotrichiellaceae* sp. UM 238, isolated from human skin scraping. The UM 238 genome was found to contain many classes of protective genes that are responsible for fungal adaptation under adverse environmental conditions.

## GENOME ANNOUNCEMENT

At least 70 genera of dematiaceous fungi are known to cause infection in humans (1), but many of these fungi have yet to be fully characterized. UM 238 is a dematiaceous fungus isolated from a patient’s skin scraping. On Sabouraud agar, it grew as a black colony with a wrinkled, verrucous, and convoluted surface, and exhibited conidia that were spindle-shaped, uniseptate, or occasionally biseptate arranged in a chain. The fungus is not identifiable by its microscopic characteristics, but with the use of molecular approaches, it was identified as a species of the genus *Herpotrichiellaceae*, within the order *Chaetothyriales* and class *Eurotiomycetes*.

The genome of UM 238 was sequenced using Illumina GA IIx technology with a 380-bp insert size library strategy. In total, 20 Gb of paired-end reads were generated. After preprocessing, 40 million clean reads (~100× coverage) were subsampled and assembled into 847 contigs using Velvet v1.1.07 (2), which were subsequently scaffolded and gap-filled into 129 scaffolds (≥1,000 bp; *N*_*50*_, 456 kb) of 218 contigs (≥200 bp) using the programs SSPACE v2.0 (3) and GapFiller v1.10 (4). The resulting draft genome is estimated to be ~28.46 Mb in size. The protein-coding genes were predicted using GeneMark-ES (5), with repeat regions masked on the scaffolds, resulting in 9,412 coding sequences (CDSs) (≥33 amino acids). A total of 6,247 (66.4%) putative functional genes were successfully annotated via BLAST similarity searches against the Swiss-Prot database.

Members of the class *Eurotiomycetes* include many rock-inhabiting fungi that form colonies on bare rock surfaces, an extremely stressful habitat (6, 7). The UM 238 genome was found to contain many genes associated with survival under extremotolerant conditions, including the key genes associated with melanin biosynthesis via the 1,2-dihydronaphthalene pathway. Melanization protects fungal cells against UV radiation (8). Also present were genes encoding UV radiation resistance, the UV excision repair RAD23 homolog (9), and UV damage endonuclease UVE-1 proteins (10). Genes associated with hyperosmotic protein 21 (Shop21) (11), osmosensor Sho1 (12), and osmotic stress-induced proline dehydrogenases 1 and 2 (13), which are required during the osmotic stress response, were also found. A notable finding was the presence of genes encoding the cold-shock proteins CspV and CspA (14), a cold-sensitive U2 snRNA suppressor 2 homolog (15), and the cold-inducible RNA-binding protein B (16). The presence of these genes may suggest that UM 238 has a cold-shock response in order to tolerate extremely cold environments. Another interesting feature noted was the identification of a few cell-division-cycle-associated genes (*CDC42*) that control cellular polarization, which is essential for meristematic growth under adverse environmental conditions (17). The meristematic growth form of fungi is morphologically very similar to the muriform cells that are the invasive form in human chromoblastomycosis. The general characteristics of adaptation to adverse environments, generation of melanin, and meristematic growth traits may have the potential to incidentally turn extremotolerant fungi, like UM238, into a human pathogen (7).

### Nucleotide sequence accession number.

The nucleotide sequences of the *Herpotrichiellaceae* sp. UM 238 genome have been deposited in DDBJ/EMBL/GenBank under the accession no. AMYF00000000.
